# Nomenclature clarification: synovial fibroblasts and synovial mesenchymal stem cells

**DOI:** 10.1186/s13287-019-1359-x

**Published:** 2019-08-19

**Authors:** Fangqi Li, Yiyong Tang, Bin Song, Menglei Yu, Qingyue Li, Congda Zhang, Jingyi Hou, Rui Yang

**Affiliations:** Department of Orthopedic, Sun Yat-sen Memorial Hospital Sun Yat-sen University, NO.107 Yan Jiang West Road, Guangzhou, Guangdong Province, 510120 China

**Keywords:** Synovium, Synoviocytes, Fibroblasts, Mesenchymal stem cells, MSCs

## Abstract

Synovial-derived cells, found in the synovial membrane of human joints, were obtained by digestion of the synovial membrane and were subsequently expanded in vitro. The identity of synovial-derived cells has long been a topic of debate. The terms “type B synoviocytes,” “fibroblast-like synoviocytes (FLS),” “synovium-derived mesenchymal stem cells (MSCs),” and “synovial fibroblasts (SF)” appeared in different articles related to human synovial-derived cells in various disease models, yet they seemed to be describing the same cell type. However, to date, there is no clear standard to distinguish these terms; thus, the hypothesis that they represent the same cell type is currently inconclusive. Therefore, this review aims to clarify the similarities and differences between these terms and to diffuse the chaotic nomenclature of synovial-derived cells.

## Background

Our group has been working on synovial-derived cells for more than 10 years. Initially, we named this group of cells as “articular synoviocytes” because these cells were derived from the joint cavity [1]. Then, we found that Barland [2] and Ghadially [3] divided the synovial intimal cells into two cell types via electron microscopy: type A cells and type B cells; thus, we renamed our cells as “type B synoviocytes” to reflect their phenotype more accurately [4]. Yet, in the study of the immune response of knee joint allogeneic tendon transplantation [5], we found that this group of cells has stem cell-like characteristics and immunomodulatory abilities, similar to De Bari’s study of synovial membrane-derived mesenchymal stem cells (MSCs) [6]. Therefore, we began to name these cells as “synovium-derived MSCs” [7–11]. However, when it comes to research concerning rheumatoid arthritis, we found that most researchers preferred to use “fibroblast-like synoviocytes (FLS)” or “synovial fibroblasts (SF)” when referring to synovial-derived cells. These findings intrigued us to explore the question: what should be the best classification of synovial-derived cells? With various nomenclature in mind, there is a need to redefine these cells to better standardize their definition.

## Introduction

**Fig. 1:**
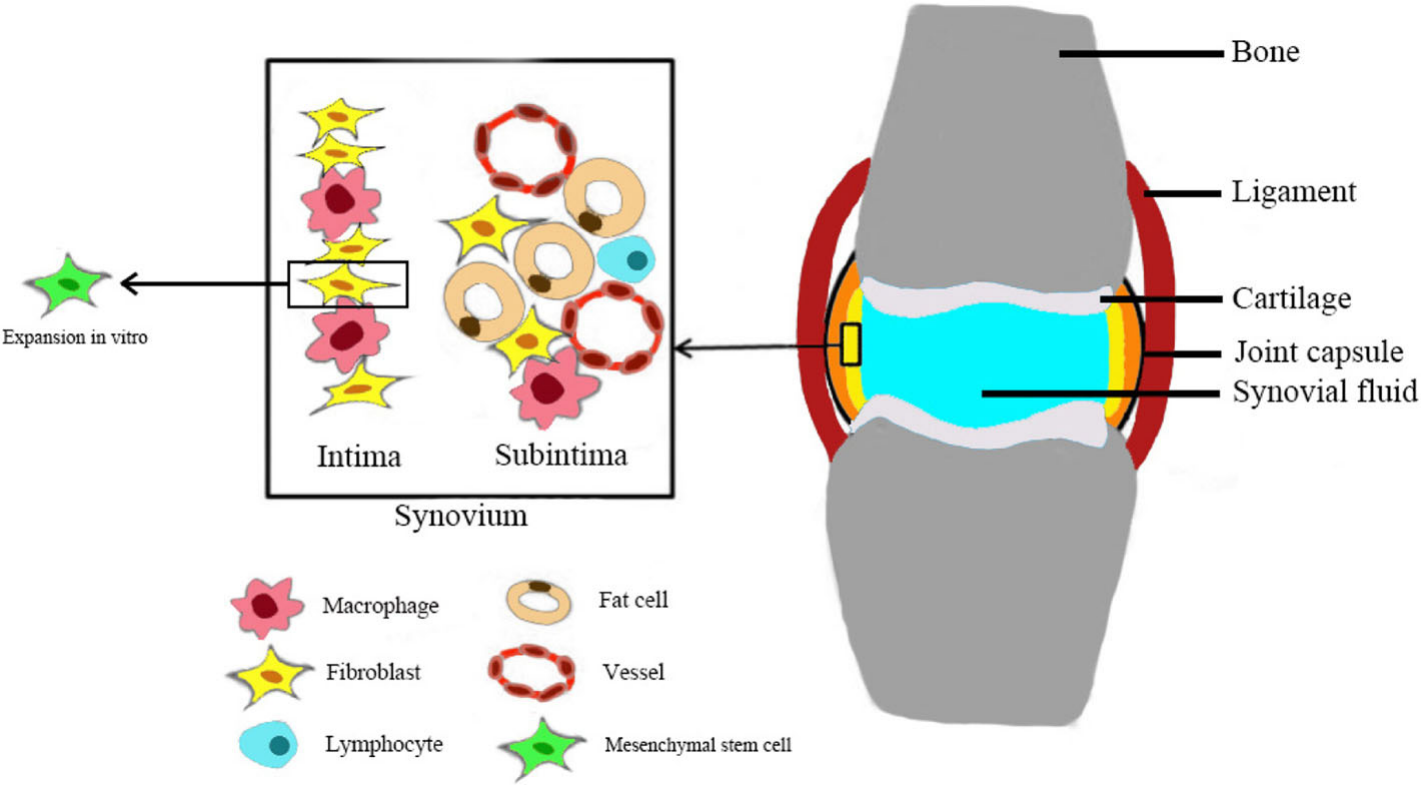
The distribution of various cells in the synovium. The term “synovial fibroblasts (SF),” which refers to intimal and subintimal fibroblasts, appears to have a broader definition as compared to “type B” or “fibroblast-like synoviocytes (FLS),” which only represents intimal fibroblasts. Synovial intimal fibroblasts express VCAM-1 and high level of UDPGD, while subintimal fibroblasts do not. Fibroblasts can be isolated from synovium and exhibit the characteristics of mesenchymal stem cells (MSCs) after in vitro culture and proliferation. Therefore, these cells are considered as synovium-derived MSCs. FLS can function as innate immune cells, expressing variety of immune regulatory cytokines. Synovium-derived MSCs have been shown to have immunosuppressive properties in vitro

The term “synovial membrane (SM)” refers to the special mesenchymal tissue lining the spaces of joints, tendon sheaths, and bursae. SM consists of two anatomically distinct layers: the continuous surface layer of cells (intima) which is 20–40 mm thick in cross-section and the underlying tissue (subintima) which can span to 5 mm in thickness. The intima consists of macrophages and fibroblasts while the subintima includes scattered blood vessels, fat cells, and fibroblasts, with few lymphocytes or macrophages (Fig. 1). MSC-like cells have also been found in the synovial membrane [12]. Synovial tissue can also be histologically classified into three regions: surface, stromal, and perivascular regions [13, 14].

While reviewing a series of past studies, we were presented with confusing nomenclature of the synoviocytes (Table 1). In 1962, Barland [2] studied the human synovial intimal cells by electron microscopy and he discovered two distinct types of intimal cells in normal synovium which he designated “type A cell” and “type B cell”. Type B cell contained large amounts of endoplasmic reticulum with fewer vacuoles and vesicles, and it was also believed to be mainly secretory, probably manufacturing components of the synovial fluid. In 1967, Ghadially studied the synovium in patients with rheumatoid arthritis (RA) and used the term “type B synovial cells” to describe the fibroblast-like cells. In 1972, Rosales [15] first used the term “synovial fibroblasts (SF)” to describe the cells derived from synovium in his study of RA and non-RA patients. He found that these cells which possess extensive rough endoplasmic reticulum were able to synthesize high molecular weight hyaluronic acid and fibrous collagen, which conforms to the characteristics of type B cells. Later, the term “synovial fibroblasts” can be commonly seen in other articles related to human synovial-derived cells and was often referred to with the acronym “RASF” in some articles related to RA [16, 17]. In 1992, Wilkinson [20] reassessed human synovial intimal cells by light microscopy and proposed that type B cells were similar to fibroblasts, but were significantly distinct from other fibroblasts by its high activity of uridine diphosphate glucose dehydrogenase (UDPGD); thus, he named these cells “fibroblast-like intimal cells”. In 1996, Firestein [26] used the term “fibroblast-like synoviocytes (FLS)” to describe type B cells. In his research on patients with RA, he proposed that the surface expression of the adhesion molecule vascular cell adhesion molecule-1 (VCAM-1) and the intracellular localization of the enzyme UDPGD were the two best-defined differences that distinguished intimal fibroblasts from subintimal fibroblasts as well as fibroblasts from other sources. In 2011, Smith [13, 14] further summarized the structure and function of the human normal synovium and used the terms “type A synoviocytes” and “type B synoviocytes” to describe two types of synovial intimal cells. Type A synoviocytes possessed cell surface markers such as CD163 and CD68, which identifies it as being from the macrophage lineage, while type B synoviocytes carried cell surface markers such as CD44 and intercellular adhesion molecule-1 (ICAM-1), which categorizes these cells into the fibroblast lineage. Therefore, Smith [13] suggested that type A synoviocytes were probably macrophages derived from blood monocytes, while type B synoviocytes were intimal fibroblasts which were locally derived.

**Table 1:** Different nomenclature of human synovium-derived cells

Nomenclature used	Tissue samples	Cell morphology	Markers	Differentiation potential	Ref.
Cell type B	Human	NA	NA	NA	[2]
Type B synovial cell	Human	NA	NA	NA	[3]
SM-derived MSCs	Human	Fibroblast-like	+: Vimentin/VCAM1/CD44 −: CD14-	Chondrogenesis/osteogenesis/myogenesis/adipogenesis	[6]
SM-derived MSCs	Human	NA	+: CD44/CD90/CD105/CD166 −: CD14/CD34/CD45/HLA-DR	Chondrogenesis/osteogenesis/adipogenesis	[9]
Synovial fibroblasts	Human	Fibroblast-like	NA	NA	[15–19]
Fibroblast-like synovial intimal cell/synoviocyte	Human	Fibroblast-like	+: UDPGD −: CD68/CD22/CD3	NA	[20]
SM-derived MSCs	Human	NA	+: CD44/CD73/CD90/CD105 −: CD14/CD34/CD45	Chondrogenesis/osteogenesis	[21]
Fibroblast-like synoviocytes	Human	NA	+: VCAM1	NA	[22]
Fibroblast-like cells	Human	Fibroblast-like	+: LBs/SP-A	NA	[23]
Synovial MSCs	Human	NA	+: CD73/CD105/CD90/CD44/CD151/CD166 −: CD45/CD31	Chondrogenesis/osteogenesis/adipogenesis	[24]
Synovial MSCs	Human	Fibroblastic spindle shape	+: CD105/CD90/CD44	Chondrogenesis/osteogenesis/adipogenesis	[25]
Fibroblast-like synoviocyte	Review	NA	NA	NA	[26–28]

*MSCs* mesenchymal stem cells, *VCAM1* vascular cell adhesion molecule 1, *UDPGD* uridine diphosphate glucose dehydrogenase, *LBs* lamellar bodies, *SP-A* surface protein A, *NA* not applicable

Mesenchymal stem cells (MSCs) were first identified by Friedenstein in 1976 from the human bone marrow [29]. These cells were identified as distinct from hematopoietic stem cells (HSCs) based on their plastic-adherent capacity. The term “MSCs” was coined by Caplan in 1991 to represent the multilineage potential of the cells [30]. In 2001, De Bari et al. first isolated MSCs from human synovial membrane (SM) and called them “SM-derived MSCs” [6]. These cells had the ability to proliferate extensively in culture, and they maintained their multilineage differentiation potential in vitro. They also showed that SM-derived MSCs appeared to be a relatively homogeneous population of fibroblast-like cells during in vitro expansion, which hinted that SM-derived MSCs were related to synovial fibroblasts. Since then, SM-derived MSCs were also called “synovium-derived MSCs” [21], or “synovial MSCs” [31], and the terms were used interchangeably. In 2006, the International Society for Cellular Therapy (ISCT) provided a set of minimal criteria to describe a cell as multipotent MSCs [32]. This includes three aspects: First, the cells must be plastic adherent when maintained under standard conditions. Second, they must express CD105, CD73, and CD90 and lack the expression of CD45, CD34, CD14 or CD11b, CD79α, or CD19, and HLA-DR surface molecules. Third, the MSCs must be able to differentiate into osteoblasts, adipocytes, and chondrocytes in vitro. Therefore, these criteria were widely used to identify SM-derived MSCs in subsequent studies. However, there has been some controversy over the term “MSCs” recently. Caplan [33], who first named the MSCs, urged that it is more accurate to change the term “mesenchymal stem cells” to “medicinal signaling cells” as MSCs do not function in the body as progenitors for tissues, either in the normal steady-state or in disease or in circumstances of disease or injury; they should not be categorized as stem cells. Additionally, Soundararajan [34] systematically compared MSCs and fibroblasts and concluded that MSCs are in fact immature fibroblasts and that the two cells were most likely to be the same type of cells. These controversies and speculations spur us to redefine the nomenclature of SM-derived MSCs and their relationship with synovial fibroblasts.

### Relationship between type B synoviocytes, FLS, and SF

#### Type B synoviocytes and fibroblast-like synoviocytes

These two names represent the same cell and can be used interchangeably. It has been proven that type B synoviocytes derived from human synovium possess the morphologic appearance of fibroblasts as well as the structural machinery to synthesize and secrete an impressive array of products, including proteoglycans, cytokines, arachidonic acid metabolites, and metalloproteinases [2, 20]. Therefore, it is generally recognized that the term “fibroblast-like synoviocytes” can be used to represent type B synoviocytes. The term “type B synoviocytes” or “type B cells” mainly appeared in early articles associated with synovial tissue and researchers simply used the letter A and B to distinguish between two types of synovial intimal cells. However, the term “fibroblast-like synoviocytes” is more commonly used in various articles, especially the articles about RA, to highlight the important role of fibroblasts in the initiation and perpetuation of destructive joint inflammation [27, 28].

#### Synovial fibroblasts and fibroblast-like synoviocytes

Synovial fibroblasts, as the name implies, are fibroblasts located in synovial tissue with the general characteristics of fibroblasts. Thus, the term “synovial fibroblasts,” which refers to intimal and subintimal fibroblasts, appears to have a broader definition as compared to “type B” or “fibroblast-like synoviocytes,” which only represents intimal fibroblasts. There is ample evidence supporting the statement that synovial intimal fibroblasts differ from subintimal fibroblasts. Human synovial intimal fibroblasts express high levels of UDPGD, which is related to the production of hyaluronan, while subintimal fibroblasts do not [20, 35]. Additional evidence would be that human synovial intimal fibroblasts expressed several adhesion molecules, including VCAM-1, ICAM-1, CD44, and β_1_ integrins [13, 22], while subintimal fibroblasts and fibroblasts from other sources only expressed lower levels of CD44 and β_1_ integrins and do not express VCAM-1 [14, 36]. Therefore, the term “synovial fibroblasts” appears to be different from “fibroblast-like synoviocytes” because the former term includes a broader definition of synovial-derived cells. This may also be the reason why some studies used the term “fibroblast-like synoviocytes” rather than “synovial fibroblasts” to describe human synovial intimal fibroblasts.

### Synovium-derived MSCs vs synovial fibroblasts

#### Origination

It is generally accepted that synovial fibroblasts originate from synovial tissue. However, there are two different views on the origin of synovium-derived MSCs. One is that MSCs are brought into the synovium via migrating blood vessels. This was proposed because MSCs are generally located around blood vessels, and the frequency of MSC is positively correlated with increasing tissue vascularity [37, 38]. The other perspective is that the synovium-derived MSCs originate from the synovial intima. Vandenabeele et al. [23] investigated the phenotypic characteristics of synovium-derived MSCs and found that both synovium-derived MSCs and type B synoviocytes contained characteristic lamellar bodies (LBs), which indicated that synovium-derived MSCs may originate from the synovial intima, as they have a phenotype highly similar to that of type B synoviocytes. Additionally, the morphology and gene expression profile of synovial fluid-derived MSCs have been shown to be similar to synovium-derived MSCs rather than bone marrow MSCs, further supporting the hypothesis of the synovial origin of these MSCs [24, 39, 40].

#### Morphology

A number of studies have demonstrated that synovial fibroblasts and synovium-derived MSCs display similar morphology in vitro and were presented as spindle shaped. For example, in our study of synovium-derived MSCs, after 2 weeks of in vitro cultivation, the cells obtained from the digestion of synovial tissue from patients with meniscus injury were mostly spindle-shaped fibroblast-like cells [8]. In another independent research, Harvanova et al. [25] cultured MSCs derived from human synovium and synovial fluid, where they discovered that spindle-shaped fibroblast-like cells became the predominant subtype in the culture after 10 days. Additionally, Prado et al. [41] cultured MSCs derived from horse synovium and synovial fluid, and they also found that the cultures yielded adherent cells with a fibroblast-like shape. Evidently, synovial fibroblasts and synovium-derived MSCs do not significantly differ in morphology.

#### Cell surface markers

Cell surface markers frequently employed to distinguish and isolate different cell types. It has been proven that synovial fibroblasts express several adhesion molecules, such as VCAM-1, CD44, and β_1_ integrins, as well as fibroblast makers, such as collagen, vimentin, and α-actin. Similarly, synovium-derived MSCs were also observed to be positive for these surface immunophenotypes [6]. According to the current criteria defined by the ISCT, MSCs are those that express CD105, CD73, and CD90 and lack the expression of CD45, CD34, CD14 or 11b, CD9α, or CD19, and HLA-DR surface molecules [32]. Interestingly, synovium-derived MSCs also showed nearly identical markers as defined by ISCT [6, 42, 43]. However, there is little evidence of whether fibroblasts from synovial tissue possess the expression characteristics of these cell surface markers, though a large body of evidence supports that fibroblasts derived from dermal and adipose tissue do have these characteristics [44, 45]. Synovium-derived cells are generally identified using a combination of non-specific markers, rather than several specific markers. Hence, it is inconclusive whether these cells are MSCs or synovial fibroblasts.

#### Proliferative capacity and differentiation potential

A major characteristic of MSCs is their ability to self-renew for a long period of time without significant changes in their properties. Although fibroblasts are generally considered to be limited by a number of mitotic divisions because of their status as differentiated cells, studies have shown that fibroblasts and MSCs exhibited very similar growth rates [46, 47]. Synovium-derived MSCs have been reported to possess high self-renewal capacity [6, 48]. De Bari et al. [6] showed that synovium-derived cells from adult human donors of various ages can be expanded in vitro over at least ten passages, with limited cell senescence. Sakaguchi et al. [48] found that the proliferative ability of synovium-derived cells was retained even after ten passages. However, these studies did not compare the synovium-derived cells to the fibroblasts derived from synovial tissue; thus, the possibility that these cells are synovial fibroblasts cannot be ruled out.

One of the other aspects of the definition of MSCs offered by the ISCT is the differentiation potential of the cells into osteocytes, adipocytes, and chondrocytes in vitro. Synovium-derived MSCs have been shown to differentiate into these three cells [6, 49, 50]. Although Alt et al. [46] found that fibroblasts exhibited no differentiation potential, other studies have shown that fibroblasts do possess the potential to differentiate into osteocytes, adipocytes, and chondrocytes [45, 47, 51]. Therefore, it is difficult to distinguish whether the synovium-derived cells are MSCs or synovial fibroblasts based on their differentiation potential.

#### Immunologic properties

Synovium-derived MSCs have been shown to have immunosuppressive properties in vitro. They have been shown to inhibit the activation and proliferation of T cells, besides being also able to induce the formation of T-reg cells [8, 21, 52]. Hagmann et al. [53] found that MSCs isolated from patients with osteoarthritis (OA) can retain the percentage of T-regs when co-cultured with T-reg-enriched lymphocytes from healthy donors, with IL-6 playing an important role in mediating these processes. Djouad et al. [21] provided the first evidence that MSCs isolated from the synovial membrane could suppress T cell response in a mixed lymphocyte reaction, and simultaneously expressing the indoleamine 2,3-dioxygenase (IDO) enzyme (possible mediator of this suppressive activity) to a similar extent as bone marrow MSCs. In our study of synovium-derived MSCs, we also confirmed that these cells have the effect of inhibiting the proliferation of T cells, and this inhibition is enhanced with increasing numbers of MSCs [8].

On the contrary, synovial fibroblasts have been mostly known to be mediators of inflammation and key players in the pathogenesis of several chronic diseases, such as rheumatoid arthritis (RA) [27]. However, there is considerable evidence that synovial fibroblasts in RA are different from normal fibroblasts [18], and this difference could contribute to the increased inflammation in organs. Interestingly, a study comparing fibroblasts and MSCs in terms of their immunomodulatory properties concluded that they both had similar properties in immunomodulation [54]. Both cell types were shown to directly suppress T cell proliferation, although the suppression by MSCs was more potent than fibroblasts [54]. In addition, both fibroblasts and synovium-derived MSCs were also known to mediate their immunoregulatory role via IDO-dependent mechanisms and TGF-β [19, 54].

#### Research and clinical use

Among the early clinical use of MSCs, the most dramatic effect was described by Le Blanc et al. [55] when they transplanted haploidentical MSCs in a 9-year-old boy with severe acute graft-versus-host disease (GvHD). Clinical response was striking, and the patient achieved complete recovery after 1 year. Thus, they postulated that MSCs possessed a potent immunosuppressive effect, which has been confirmed in subsequent studies. Moreover, synovium-derived MSCs have also been shown to have potent immunosuppressive effects [8, 21, 52]. Therefore, synovium-derived MSCs have gradually become a hotspot in the field of transplantation. For example, Kubosch et al. [56] found that synovium-derived MSCs possessed great chondrogenic potential and showed encouraging results for cartilage repair purposes. Nakagawa et al. [57] found that the transplantation of synovium-derived MSCs could promote healing after meniscal repair.

## Conclusion

Both “type B synoviocytes” and “fibroblast-like synoviocytes (FLS)” can represent the synovial intimal cells and can be used interchangeably. The term “synovial fibroblasts (SF)” can also represent the synovial cells, with a broader definition, encompassing both the intimal and subintimal fibroblasts.

As for the term “synovium-derived MSCs,” there might be two different implications of its nomenclature. One implication is that cells isolated from synovial tissue possessed characteristics of MSCs, so these cells are called “synovium-derived MSCs.” However, most articles identifying these cells rely on morphology and surface markers, which have been observed to be very similar to synovial fibroblasts. This makes it very difficult to conclusively identify whether the isolated or analyzed cells in a given study are synovial fibroblasts or synovium-derived MSCs. The other implication is that MSCs are probably immature fibroblasts. This suggests that synovial fibroblasts are likely derived from synovial MSCs. More experiments are needed to prove this implication. All in all, the MSCs that we isolated and cultured from the synovium were most likely fibroblasts. However, we were unable to distinguish whether these synovial-derived cells were fibroblasts or MSCs by cell morphology, cell surface markers, differentiation potential, and immunomodulatory properties. Therefore, although SM-derived MSCs and synovial fibroblasts are likely to refer to the same cell type, the available evidence is insufficient and further studies are needed to better clarify the relationship between the two.

## Data Availability

Data sharing is not applicable to this article as no datasets were generated or analyzed during the current study.

## Abbreviations

FLS: Fibroblast-like synoviocytes
GvHD: Graft-versus-host disease
HSCs: Hematopoietic stem cells
ICAM-1: Intercellular adhesion molecule-1
IDO: Indoleamine 2,3-dioxygenase
ISCT: International Society for Cellular Therapy
LBs: Lamellar bodies
MSCs: Mesenchymal stem cells
OA: Osteoarthritis
RA: Rheumatoid arthritis
SF: Synovial fibroblasts
SM: Synovial membrane
UDPGD: Uridine diphosphate glucose dehydrogenase
VCAM-1: Vascular cell adhesion molecule-1
